# Multi-organ imaging demonstrates the heart-brain-liver axis in UK Biobank participants

**DOI:** 10.1038/s41467-022-35321-2

**Published:** 2022-12-21

**Authors:** Celeste McCracken, Zahra Raisi-Estabragh, Michele Veldsman, Betty Raman, Andrea Dennis, Masud Husain, Thomas E. Nichols, Steffen E. Petersen, Stefan Neubauer

**Affiliations:** 1 Division of Cardiovascular Medicine, Radcliffe Department of Medicine, University of Oxford, National Institute for Health Research Oxford Biomedical Research Centre, Oxford University Hospitals NHS Foundation Trust, Oxford, OX3 9DU UK; 2 William Harvey Research Institute, NIHR Barts Biomedical Research Centre, Queen Mary University of London, Charterhouse Square, London, EC1M 6BQ UK; 3 Barts Heart Centre, St Bartholomew’s Hospital, Barts Health NHS Trust, West Smithfield, London, EC1A 7BE UK; 4 Wellcome Centre for Integrative Neuroimaging (WIN FMRIB), University of Oxford, Oxford, UK; 5 Department of Experimental Psychology, University of Oxford, Oxford, UK; 6 Perspectum Ltd, Gemini One, 5520 John Smith Drive, Oxford, OX4 2LL UK; 7 Nuffield Department of Clinical Neurosciences, University of Oxford, Oxford, UK; 8 Nuffield Department of Population Health, Oxford Big Data Institute, Li Ka Shing Centre for Health Information and Discovery, University of Oxford, Oxford, UK; 9 Health Data Research UK, London, UK; 10 The Alan Turing Institute, London, UK

**Keywords:** Biomarkers, Risk factors, Epidemiology

## Abstract

Medical imaging provides numerous insights into the subclinical changes that precede serious diseases such as heart disease and dementia. However, most imaging research either describes a single organ system or draws on clinical cohorts with small sample sizes. In this study, we use state-of-the-art multi-organ magnetic resonance imaging phenotypes to investigate cross-sectional relationships across the heart-brain-liver axis in 30,444 UK Biobank participants. Despite controlling for an extensive range of demographic and clinical covariates, we find significant associations between imaging-derived phenotypes of the heart (left ventricular structure, function and aortic distensibility), brain (brain volumes, white matter hyperintensities and white matter microstructure), and liver (liver fat, liver iron and fibroinflammation). Simultaneous three-organ modelling identifies differentially important pathways across the heart-brain-liver axis with evidence of both direct and indirect associations. This study describes a potentially cumulative burden of multiple-organ dysfunction and provides essential insight into multi-organ disease prevention.

## Abstract (web summary)

While heart disease, dementia and liver disease often co-occur, multi-organ imaging is needed for deeper elucidation of these cross-organ links. Here, the authors use image-derived phenotypes to describe underlying associations between heart, brain and liver health in a large population cohort.

## Introduction

Early detection and prevention of chronic non-communicable diseases is a major public health challenge. It is now well-established that many such illnesses, for example, ischaemic heart disease, stroke, neurocognitive decline and fatty liver disease tend to co-occur^1–3^, and that these conditions are critically influenced by a set of shared risk factors such as elevated body mass index (BMI), hypertension, and diabetes mellitus^4,5^. Consequently, it is no surprise that a multi-organ approach to disease prevention is an area of growing research interest^6,7^, with the heart-brain-liver axis as a potentially worthy target. While there is now extensive evidence that heart, brain, and liver disease outcomes are empirically linked^8–10^, the underlying mechanisms are not well understood.

Evolving research in medical imaging continues to produce increasingly powerful diagnostic biomarkers, some of which have been shown to precede disease^11–13^. However, existing research linking heart, brain, and liver health has rarely incorporated imaging biomarkers across multiple organs, rather using single-organ imaging alongside subjective measures such as clinical diagnoses or cognitive function tests. Where multi-organ imaging has been used, this is typically conducted within small samples or clinical cohorts with significant disease. It is not known whether individual associations identified in small clinical studies still hold in the healthy population at large, or whether multiple cross-organ associations persist when modelled simultaneously. It is important that these relationships are examined in population cohorts without severe disease, since the targets for preventative strategies are those who are clinically healthy, but may be at risk for future potential disease.

Existing work provides extensive evidence that risk factors such as diabetes and hypertension are important drivers across all three organs, but there is very little known about the multi-organ relationships independent of these primary shared determinants.

**Fig. 1:**
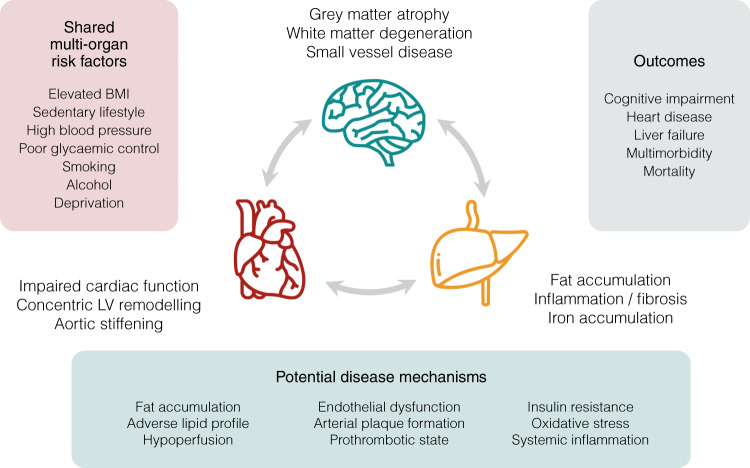
Conceptual overview of the heart-brain-liver axis. Heart, brain and liver systems co-exist within a context of shared multi-organ risk factors. In this study, we identify the ways that subclinical changes in each organ associate with others in the heart-brain-liver axis, relevant to a range of possible underlying disease mechanisms. A detailed understanding of multi-organ interaction could potentially improve our ability to predict and prevent connected complex outcomes. BMI body mass index, LV left ventricular. Please note, this is a conceptual figure and not all elements displayed are explicitly tested in this study. Image attributes: heart made by Smashicons, liver made by Vitaliy Gorbachev, brain made by Justicon, all from www.flaticon.com.

The UK Biobank is a large-scale biomedical research database that combines detailed demographic, lifestyle, and clinical characterisation with multi-organ magnetic resonance imaging (MRI), providing the ideal platform for the consideration of multi-system health interactions in a population cohort. This offers an unprecedented opportunity to combine a range of imaging metrics, old and new, and to explore what these have to tell us about the complex interplay between the heart, brain and liver organ systems (Fig. 1).

In this study, we map the relationships between organ-level imaging measures of the heart, brain and liver using state-of-the-art MRI phenotypes in 30,444 UK Biobank participants, considering a wide range of confounders and potential disease mechanisms. We provide large-sample validation for known heart-brain-liver relationships, describe some novel associations, and find evidence for differential cross-organ dependencies. We demonstrate how shared associations could point towards shared pathology and have the potential to inform multi-system risk prediction and treatment strategies.

## Results

### Study sample

**Fig. 2:**
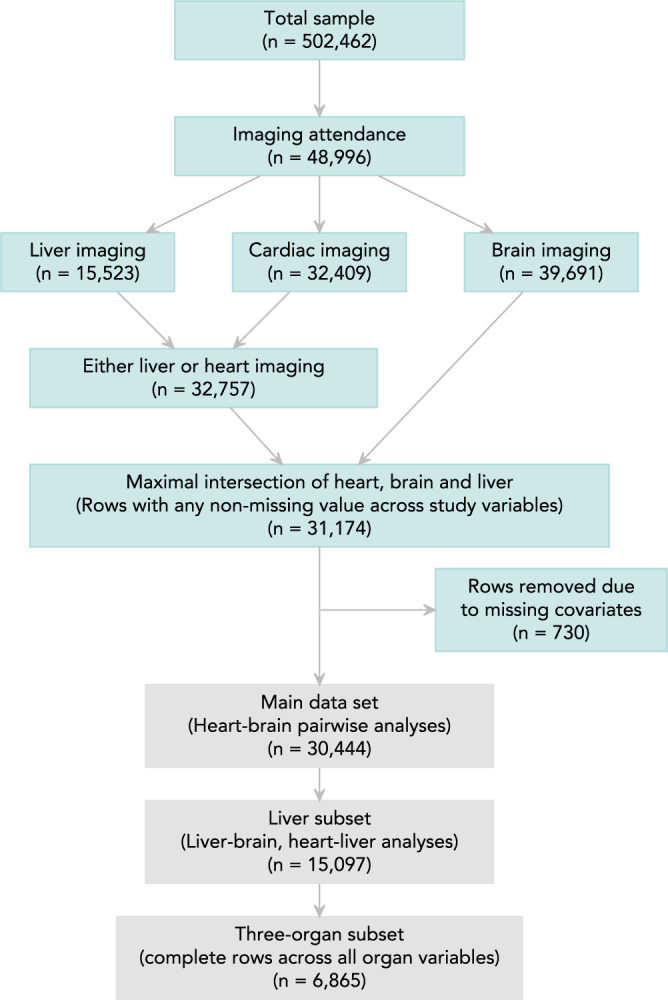
Study sample. Across the UK Biobank cohort, 48,996 participants had a record of an imaging attendance of any type. Relevant brain imaging data was available for 39,691 participants, cardiac imaging was available for 32,409 participants, and liver imaging was available for 15,523 participants. The maximum possible set at the intersection of brain imaging plus either liver or heart imaging contained 31,174 participants. We excluded 730 (2%) participants with missing covariate data, leaving a main set of 30,444 participants. This set was used for the pairwise heart-brain analyses. Within the main set, a subset of 15,097 participants had at least one piece of liver imaging data. This is the liver subset and this forms the basis of the pairwise liver-brain and liver-heart analyses. Even so, there is still variation in sample sizes across heart, brain and liver imaging metrics, described in detail in Supplementary Table 1. The data set with complete case data available across all three organs has 6,865 participants. This is the set that was used for the three-organ path analyses.

Within the UK Biobank cohort, heart and brain imaging was available for 31,174 participants. We excluded 730 (2%) participants with missing covariate data, leaving a maximum analysis sample of 30,444 participants (Fig. 2). In this study, there are three nested data sets. The pairwise heart-brain analyses are based on the main set of 30,444 participants. A subset of 15,097 participants had at least one piece of liver imaging data. We refer to this as the liver subset and this forms the basis of the pairwise liver-brain and liver-heart analyses. Even so, there is remaining variation in sample sizes across heart, brain and liver imaging metrics, described in detail in Supplementary Table 1. In the three-organ path analyses, we use only complete rows across all imaging metrics, which includes 6865 participants referred to as the three-organ subset.

### Population characteristics

Our sample included 15,905 female participants (52.2%) and 14,539 male participants (47.8%). The average age was 63.2 (±7.5) years, with median BMI of 25.9 kg/m^2^ (Table 1). The proportion of participants with diabetes, hypertension and BMI ≥ 30 kg/m2 was 5.7%, 32.4%, and 18.0%, respectively (Table 1). The three study samples (main set, liver subset and the three-organ set) were similar in terms of age, sex, average blood pressure, alcohol intake, smoking and physical activity. Participants in the three-organ set (*n* = 6865) had slightly lower levels of diabetes, hypertension and elevated BMI than the maximum set (4.9%, 29.8% and 15.3%, respectively, Table 1).

**Table 1:** Sample characteristics

Characteristic	Main set (*n* = 30,444)	Liver subset (*n* = 15,097)	Three-organ set (*n* = 6865)
Female	15,905 (52.2%)	8,145 (54.0%)	3,736 (54.4%)
Male	14,539 (47.8%)	6,952 (46.0%)	3,129 (45.6%)
Age at imaging (years)	63.2 (±7.5)	62.6 (±7.5)	62.7 (±7.4)
SBP (mmHg)	137.8 (±18.2)	137.0 (±17.9)	136.8 (±17.3)
BMI (Kg/m2)	25.9 [23.5, 28.8]	25.7 [23.4, 28.5]	25.7 [23.5, 28.4]
BMI ≥ 30 kg/m^2^	5469 (18.0%)	2446 (16.2%)	1052 (15.3%)
Smoking status			
Never smoked	18,962 (62.3%)	9462 (62.7%)	4346 (63.3%)
Previous smoker	10,160 (33.4%)	4953 (32.8%)	2232 (32.5%)
Current smoker	1095 (3.6%)	566 (3.7%)	241 (3.5%)
Physical activity (total MET-minutes/week)			
Highly active (>3,000)	9635 (31.6%)	4557 (30.2%)	2082 (30.3%)
Moderately active (600–2,999)	15,551 (51.1%)	7833 (51.9%)	3605 (52.5%)
Inactive (<600)	5008 (16.4%)	2573 (17.0%)	1126 (16.4%)
(Missing)	250 (0.8%)	134 (0.9%)	52 (0.8%)
Alcohol consumption frequency			
Never	1963 (6.4%)	955 (6.3%)	418 (6.1%)
Less than once per week	6547 (21.5%)	3212 (21.3%)	1453 (21.2%)
Once per week or more	21,704 (71.3%)	10,814 (71.6%)	4948 (72.1%)
(Missing)	230 (0.8%)	116 (0.8%)	46 (0.7%)
Diagnosed status at imaging			
Diabetes	1737 (5.7%)	753 (5.0%)	336 (4.9%)
Hypertension	9855 (32.4%)	4649 (30.8%)	2043 (29.8%)
High cholesterol	10,541 (34.6%)	5048 (33.4%)	2280 (33.2%)
Cardiac imaging			
LVSVi (ml/m2)	46.9 (±8.4)	46.8 (±8.0)	46.8 (±7.9)
LV GFI (%)	47.6 (±6.9)	47.8 (±6.7)	48.0 (±6.6)
LVM/LVEDV (g/ml)	0.57 [0.52, 0.63]	0.57 [0.52, 0.63]	0.57 [0.52, 0.63]
Aortic distensibility (x10^−3^ mmHg^−1^)	2.23 [1.60, 3.03]	2.27 [1.62, 3.07]	2.24 [1.61, 3.04]
(Missing all cardiac metrics)	581 (1.9%)	443 (2.9%)	–
Liver imaging			
Liver fat (PDFF, %)	2.87 [2.00, 4.76]	2.87 [2.00, 4.76]	3.02 [2.12, 5.22]
Fatty liver (PDFF >5%)	3503 (23.2%)*	3503 (23.2%)	1825 (26.6%)
Liver cT1 (ms)	693 [662, 729]	693 [662, 729]	691 [661, 727]
Liver iron (mg/g)	1.22 [1.13, 1.36]	1.22 [1.13, 1.36]	1.22 [1.12, 1.36]
(Missing all liver metrics)	15,347 (50.4)	–	–
Brain imaging			
Total brain volume (mL)	1498.4 (±72.9)	1504.4 (±72.6)	1502.3 (±71.9)
Grey matter volume (mL)	794.1 (±47.8)	797.3 (±47.9)	797.4 (±47.0)
White matter hyperintensities (mL)	2.74 [1.48, 5.57]	2.58 [1.40, 5.08]	2.68 [1.46, 5.17]
(Missing all brain metrics)	1,317 (4.3%)	1,317 (8.7%)	–

Entries are either count (percent) or mean (± standard deviation) or median [25th percentile, 75th percentile]. * Fatty liver percentage shows the percent of non-missing participants. *BMI* body mass index, *MET* metabolic equivalent task, *SBP* systolic blood pressure, *HbA1c* glycated haemoglobin, LVSVi left ventricular stroke volume indexed to body surface area, *LVEF* left ventricular ejection fraction, *LV GFI* left ventricular global function index, *LVM/LVEDV* left ventricular mass to volume ratio (left ventricular mass / left ventricular end diastolic volume), *PDFF* proton density fat fraction, *cT1* corrected T1 relaxation time. Source data are provided as a Source Data file.

### Associations between liver and brain indices

**Fig. 3:**
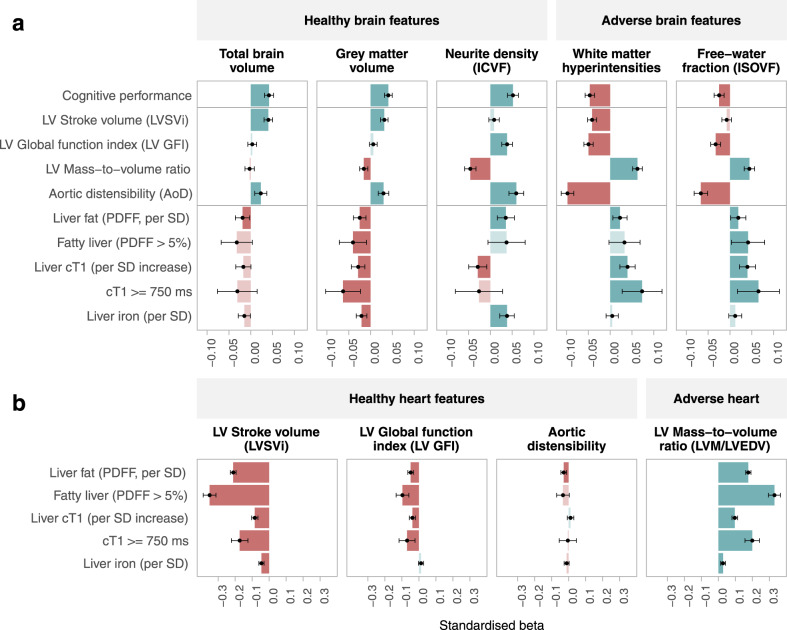
Pairwise associations between imaging phenotypes of the heart, brain and liver. Size of the bars and interval centre dot represent standardised beta coefficients, error bars represent 95% confidence intervals for the standardised beta coefficient from multivariable linear regression models. Red bars reflect negative associations, green bars reflect positive associations. **a** Heart and liver imaging metrics in pairwise associations with brain imaging metrics. **b** Pairwise associations between liver and heart imaging metrics. Models are adjusted by age, sex, diabetes, hypertension, BMI ≥ 30 kg/m2, high cholesterol, smoking, physical activity, alcohol consumption, deprivation, educational level, red blood count, total cholesterol, and glycosylated haemoglobin. Brain analyses are additionally adjusted by head size, imaging site, scanner coordinates and date of scanning. Each bar is from a separate model. Coefficient significance is assessed with a two-sided T-test, and *p*-values were considered significant after adjustment for multiple testing with a 5% false discovery rate. Where the p-value is not significant, the bar is shown in transparent colour. PDFF = proton density fat fraction, cT1 = corrected T1 relaxation time, ICVF = intracellular volume fraction, ISOVF = isotropic volume fraction, LVSVi = left ventricular stroke volume indexed to body surface area, LV GFI = left ventricular global function index, LVM/LVEDV = left ventricular mass to volume ratio (left ventricular mass / left ventricular end-diastolic volume), AoD = aortic distensibility. Associations between cognitive performance and brain imaging were calculated across a minimum of 25,280 participants. Liver-brain associations are from a minimum of 9649 participants, heart-brain associations are from a minimum of 20,610 participants, liver-heart associations are from a minimum of 8234 participants. Precise sample sizes for each pairwise result are provided in Supplementary Tables 2, 3 and 4. Source data are provided as a Source Data file.

In fully adjusted models, greater liver fibro-inflammation (cT1) was associated with significantly smaller grey matter brain volumes, larger volumes of white matter hyperintensities, and adverse white matter microstructure (Fig. 3a, Supplementary Table 2). Specifically, higher levels of liver fibro-inflammation (higher cT1) were associated with lower neurite density (lower ICVF) and a greater proportion of free-moving water (higher ISOVF). In sense checks against cognitive function, higher values across the healthy brain features (total brain volume, grey matter volume and neurite density, ICVF) were associated with better cognitive performance, while the reverse was true for adverse brain features (white matter hyperintensities and free-water fraction, ISOVF). Greater liver iron was associated with smaller total brain and grey matter volumes, but was positively associated with neurite density (higher ICVF).

In fully adjusted models, greater liver fat (higher PDFF) was significantly associated with adverse macroscale structural brain changes; specifically, smaller total brain and grey matter brain volumes and larger white matter hyperintensity regions. However, the associations between brain microstructure and liver fat were mixed, in that higher PDFF was associated with both higher free-water fraction (ISOVF) and higher neurite density (ICVF). In participants with PDFF above the 5% threshold, the associations with ICVF metrics were attenuated and those with ISOVF were strengthened (Fig. 3a, Supplementary Table 2).

### Associations between heart and brain indices

We found associations between adverse brain and cardiac image-derived phenotypes (Fig. 3a, Supplementary Table 3). Specifically, smaller total brain volumes, smaller grey matter volumes, and greater white matter hyperintensities were linked to significantly poorer LV function (lower LVSV), more concentric LV remodelling pattern (higher LVM/LVEDV), and lower aortic distensibility. Similarly, lower neurite density (ICVF) and a higher proportion of free-moving water (lower ISOVF) were linked to adverse cardiac phenotypes (lower LV GFI, higher LVM/LVEDV, lower aortic distensibility), with aortic distensibility showing the strongest relationships.

### Associations between liver and heart indices

In fully-adjusted pairwise models, higher values of all three liver features (PDFF, cT1, liver iron) were associated with poorer cardiac structure and function metrics (Fig. 3b, Supplementary Table 4), with the strongest signal observed for liver fat (PDFF). Higher liver fibro-inflammation (higher cT1), greater fat content (PDFF >5%), and higher liver iron were all associated with lower LVSVi, and more concentric LV remodelling patterns (higher LVM/LVEDV). Greater liver fat (higher PDFF) was associated with aortic stiffening (lower aortic distensibility), whereas the relationship between liver cT1 and aortic distensibility was not significant. In simultaneous modelling (Supplementary Table 5), higher liver fat (PDFF) had significant adverse associations with all heart features, and had a stronger effect on the heart (larger coefficient) than liver cT1 or liver iron. The heart imaging metrics most strongly associated with liver fat were reduced LV stroke volume and increased concentric LV remodelling (higher LVM/LVEDV).

### Multi-organ models

**Fig. 4:**
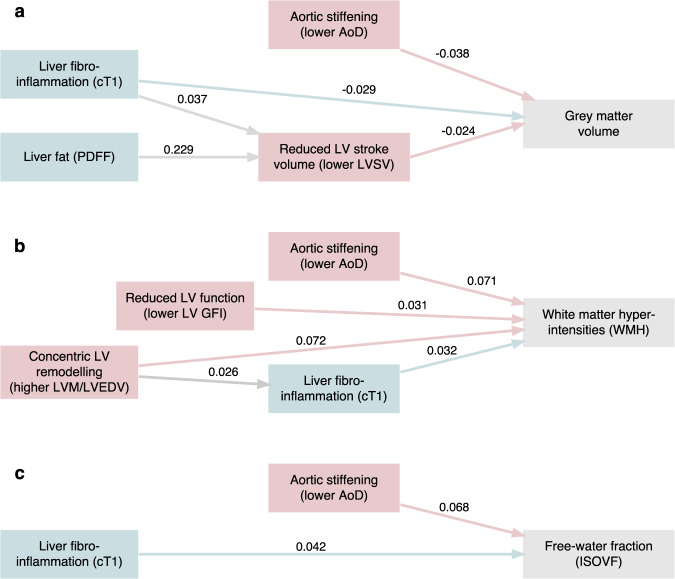
Simultaneous liver/heart associations with brain health measures. Final models and standardised beta coefficients from path analyses fitted using the *lavaan* package in R. All path analysis models are calculated with 6865 participants. **a** Simultaneous heart and liver associations with grey matter volume. **b** Simultaneous heart and liver associations with accumulation of white matter hyperintensities. **c** Simultaneous heart and liver associations with increased free-water fraction. Brain measures are in grey, heart measures are in pink and liver measures are in teal. Models are built for a single brain outcome, and initially include two liver variables as predictors (liver PDFF and cT1) and all heart variables together in two alternative forms (i) Brain ~ Heart + Liver, Heart ~ Liver, and (ii) Brain ~ Heart + Liver, Liver ~ Heart (see Supplementary Fig. 1 for detail). Non-significant paths were removed one at a time to reach the final model, then the best fitting form based on highest Tucker-Lewis fit index and lowest root mean squared error. All paths are adjusted by age, sex, height, diabetes, hypertension, high cholesterol, smoking, physical activity, alcohol intake frequency, Townsend deprivation score, education, systolic blood pressure, BMI ≥ 30 kg/m^2^, total cholesterol, glycated haemoglobin, red blood cell count. Paths connecting to the brain are additionally adjusted by imaging confounders. Coefficient significance was assessed with a two-sided Z-test, and p-values were considered significant after adjustment for multiple testing with a 5% false discovery rate. Prior to simultaneous modelling, liver and heart variables were orthogonalized to remove within-organ correlation (see Supplementary Table 4 for details). LV = left ventricular, PDFF = proton density fat fraction, cT1 = corrected T1 relaxation time, ISOVF = isotropic volume fraction, AoD = aortic distensibility. Source data are provided as a Source Data file.

Three-organ path analysis modelling found direct and indirect associations with both heart and liver for all three brain outcomes (Fig. 4, Supplementary Table 6). Aortic stiffening (lower aortic distensibility) and liver cT1 were the strongest heart and liver features respectively, both directly associated with smaller grey matter volumes, greater white matter hyperintensities and higher ISOVF, even after adjustment for confounders. For all three brain outcomes, aortic stiffening had a stronger signal than liver cT1. In addition to the features mentioned above, smaller grey matter volumes were also associated with reduced LVSVi, which itself was strongly associated with higher liver fat. On the other hand, the accumulation of white matter hyperintensities was associated with greater concentric LV remodelling (higher LVM/LVEDV) and reduced LV global function index, in addition to aortic stiffening and liver cT1. While the variance explained in brain outcomes (R^2^) was comparable between alternative model forms (see Fig. 2), the final three-organ models reported in Fig. 4 had better overall fit (lower root mean squared error, higher Tucker-Lewis index) than their alternative form counterparts (see Supplementary Table 6 for full information).

## Discussion

### Summary of findings

In this large population-based cohort of 14,539 men and 15,905 women from the UK Biobank, we present multiple patterns of association across the heart-brain-liver axis using multi-organ imaging. Greater liver fat, higher liver fibro-inflammation, and greater liver iron were linked to smaller total brain and grey matter volumes, larger volumes of brain microvascular injury, and poorer white matter microstructure (diffusion metrics). Similar associations were observed between brain and heart imaging metrics. Adverse liver features were linked to unhealthy cardiovascular structure and function (poorer LV function, more concentric LV remodelling and lower aortic compliance). The most consistent markers in the liver-brain, liver-heart, and heart-brain associations were liver fibro-inflammation (cT1), liver fat (PDFF), and aortic distensibility, respectively. Importantly, these relationships were independent of a wide range of shared demographic, lifestyle, environmental, and vascular risk factors such as diabetes mellitus, hypertension, high cholesterol, elevated BMI and smoking. Simultaneous path analysis illustrated both direct and indirect relationships between the three organ systems, further supporting interdependencies of the heart-brain-liver axis.

### Liver and brain associations

Liver tissue cT1 is elevated in association with increased inflammation and fibrosis, and as such is considered a marker of liver fibro-inflammation^14^. We demonstrated that liver fibro-inflammation (higher cT1) is the primary way that the liver associates with adverse brain features, namely decreased brain volume, increased white matter hyperintensities, lower neurite density (ICVF) and higher free-water fraction (ISOVF).

Previous studies in smaller cohorts have connected fatty liver disease status with brain atrophy^15–18^ and greater white matter hyperintensity burden^19–21^. While we confirmed these findings, however, when it comes to the microscale imaging metrics, we found the relationship between liver fat and white matter to be more nuanced. Our findings indicated that within the lower/healthy range (0 – 4.9%), liver fat was associated with higher neurite density. This unusual observation may be related to a phenotypic expression for liver fat in the normal range in some people^22,23^, or may reflect the role of fatty acids in the formation and maintenance of axonal myelin, itself composed of 70–85% lipids^24^. It is important to note, however, that when liver fat levels exceeded the 5% threshold, the potentially positive brain effects disappeared, while adverse brain effects became prominent (for example, greater ISOVF). In simultaneous modelling, liver fat did not associate directly with adverse brain phenotypes when liver cT1 was present in the model, pointing to fibroinflammation rather than liver fat as the key driver of the relationship. On the other hand, most participants in UK Biobank are within the healthy liver fat range, therefore our results may not adequately capture those with very high liver fat, meaning that these relationships may be different in other less healthy cohorts.

### Heart and brain associations

We report clear associations between the heart and brain across all structural and functional metrics considered, independent of known shared risk factors. Aortic distensibility showed the strongest associations, consistent with several smaller previous studies linking aortic stiffness and brain ageing^25–27^. Our observations linking poorer LV function and reduced arterial compliance with adverse brain features provide support for the vascular hypoperfusion paradigm which links cerebral hypoperfusion to brain atrophy and microvascular plaque accumulation^28^. An earlier analysis of the UK Biobank demonstrated an association of lower LV ejection fraction with adverse structural brain alterations (lower grey matter volume and greater white matter hyperintensities)^29^. We significantly extend these observations by examining associations with a wider range of both cardiovascular and brain features including both structural and functional metrics.

Existing research connecting imaging-derived cardiac metrics to white matter microstructure is sparse. One small study (n = 318) found that increased LV mass was associated with compromised white matter in several brain areas, including the superior frontal gyrus, anterior corona radiata and superior corona radiata^30^. The current study presents associations between multiple imaging-derived heart metrics and state-of-the-art metrics of neuronal microstructure, revealing a consistent pattern of mutual support between more favourable heart and brain imaging metrics, with the strongest signal via aortic distensibility. While previous studies have connected vascular risk factors such as hypertension or smoking to brain atrophy, white matter hyperintensities and white matter microstructure^31–35^, we observed significant associations between brain and heart metrics even after adjusting for shared vascular risk factors.

### Liver and heart associations

We observed a consistent pattern of association between the liver and heart across all imaging metrics considered. The most convincing associations were observed between liver fat and the heart, with higher PDFF strongly linked to reduced LV stroke volume and more concentric LV remodelling, a pattern which has been connected to poorer mortality and clinical outcomes^36^. Although previous work has linked clinically diagnosed fatty liver disease to poorer heart health, and LV hypertrophy in particular^37^, our study validates this relationship at the organ tissue level, in a large population-based sample mostly free from clinical disease. Greater liver fibro-inflammation (cT1) and higher liver iron were also linked to adverse cardiac features, but these were less dominant than the liver fat associations. In this context, it is worth considering that, in many individuals, elevated liver fat precedes liver fibrosis, and it is possible that the stronger association of measures of cardiac health with liver fat rather than fibro-inflammation is simply due to the fact that in the UK Biobank population, changes in fat are of a greater magnitude than changes in fibro-inflammation. Hence, it is possible that associations of heart health with liver fibro-inflammation may become more dominant in a population with more advanced liver disease.

### Three-organ network

In addition to pairwise associations, simultaneous three-organ path analysis revealed some potentially important aspects of the heart-brain-liver axis. Among the heart and liver features studied, two factors emerged as dominant in their association with brain health – namely aortic distensibility and liver fibroinflammation (cT1). These features associate consistently with adverse brain alterations at the macro- and micro-scale. These findings would suggest that therapies aimed at improving vascular health and reducing liver inflammation may have additional positive benefits for the brain. In addition to these direct effects, there is evidence of interplay between the liver and heart, potentially compounding adverse associations with the brain. For example, increased liver fat is associated with reduced LV stroke volume which then associates with reduced grey matter volume. Although the effect sizes were small, the observation of both direct and indirect effects provides evidence of a potentially cumulative burden of multiple-organ dysfunction.

### Strengths and limitations

UK Biobank provides multi-organ imaging acquired and analysed using standardised approaches, thereby providing an ideal platform for the present study. As we used imaging measures of organ structure and function, our assessments were not susceptible to biases and subjectivity of clinical diagnoses or cognitive function testing used in previous work. Image analysis in our study was with previously validated fully automated quality-controlled tools, minimising risk of bias. The deep characterisation of the UK Biobank cohort permitted adjustment for a wide range of potential confounders. However, there may be imperfections in the measurement of these covariates (e.g. physical activity is based on self-report), resulting in residual confounding. The analysis in this study represents cross-sectional relationships. In our three-organ models, we have assigned brain metrics as model outcomes. This is not a comment on the direction of the relationships, which we believe is most likely bidirectional, but rather for consistency and ease of assimilation. Furthermore, the three-organ model structures presented are not exhaustive of all possible combinations, but serve to highlight the most prominent simultaneous heart-brain-liver associations among the metrics studied. In future works, other approaches may be explored, directed at examining more specific research questions. Our model structures are based on the assumption that clinical diagnoses drive organ-level remodelling represented by the imaging metrics; for example, hypertension leading to LV hypertrophy. We believe that this is the most likely direction of these relationships and that therefore the clinical diagnoses are confounder variables in the model. However, we cannot exclude the possibility that alterations in imaging metrics also drive clinical diagnoses, in which case the diagnoses would be colliders. We think that this is a less likely possibility, but one that merits further study in future work.

The UK Biobank imaging study is unique in including multi-organ imaging data for a large population cohort, permitting analyses of large samples of mostly healthy people. Available evidence supports the technical and clinical validity of the image-derived phenotypes used in our study. Evidence is accumulating demonstrating the clinical validity and trends of the CMR-derived metrics in the UK Biobank^38^. With regard to the brain imaging metrics, we have provided a “sense check” against cognitive function to aid interpretation of the direction of health. A summary of relevant published evidence for the validity of liver imaging metrics is provided in Supplementary Table 7. Overall, further research is essential in shaping a more complete understanding of the clinical validity of these measures in population cohorts. Finally, due to the observational nature of the study, we cannot exclude residual confounding or infer causation from the results.

In conclusion, we have provided an overview of the connections between the heart, brain and liver, and demonstrated that many of the individual cross-organ associations identified in smaller clinical studies can also be found in a large (mostly healthy) population sample. We have shown that multi-organ relationships persist after adjustment for dominant shared risk factors, and that multiple cross-system associations can simultaneously coexist within the same participants. Our work adds to the growing body of multi-organ evidence suggesting that adverse features in one organ (the liver, for example), may have additional implications for other organs. In this context, physicians should be aware of the ways that other organs could be at risk, and the treatments that could have potential multi-system benefit.

Because this is a hypothesis-generating study, further mechanistic studies are warranted to investigate the multi-organ impact of known risk factors (eg. diabetes, hypertension) and the pathophysiological processes underpinning the associations identified (whether vascular, inflammation, nutrition, infection or toxin-related). A more detailed examination of multi-organ mechanisms would help identify potential therapeutic strategies to maximise multi-organ benefits (e.g. statins, angiotensin-converting enzyme inhibitors, blood-sugar lowering medications).

Looking to the future, an understanding of multi-organ interdependence is fundamental to population-level risk stratification and disease prevention, since the detection of abnormalities in any of the three organs signals an opportunity to intervene earlier in patients and alter the trajectory of disease development. In summary, the cumulative potential insights from multi-system imaging are expected to have a major impact on our ability to predict and prevent complex diseases, and reliably improve quality of life and overall survival.

## Methods

### Study population and setting

This study complies with the Declaration of Helsinki; the work was covered by the ethical approval for UK Biobank studies from the National Health Service (NHS) National Research Ethics Service on 17th June 2011 (Ref 11/NW/0382) and extended on 18 June 2021 (Ref 21/NW/0157) with written informed consent obtained from all participants.

UK Biobank is a large prospective research cohort, covering approximately 500,000 population-based participants in the UK. Individuals aged 40–69 years old were identified from National Health Service (NHS) registers and invited to participate. All participants completed a baseline visit, where a range of physical measurements were taken along with a touchscreen questionnaire and in-person interview. Individuals who were not able to complete baseline assessments due to poor health or discomfort were not recruited.

### The UK Biobank Imaging Study

The UK Biobank dataset has been augmented with the addition of multi-organ imaging for a large subset of the original participants. To date, approximately 50,000 participants have completed the standardised UK Biobank imaging protocol, which includes MRI of the brain, heart, and liver. Image acquisition was in accordance with pre-defined standard operating procedures using uniform equipment and staff training. The UK Biobank imaging protocol is detailed in published sources^39–41^. A detailed description of MRI acquisition parameters for heart, brain, and liver scanning is presented in Supplementary Table 8. Recruitment for participants in this study took place between March 2006 and October 2010 with imaging between May 2014 and March 2019.

### Liver imaging-derived metrics

Liver scans were performed using a Siemens 1.5 T scanner as part of the UK Biobank abdominal imaging protocol^42^. A single transverse slice was taken through the centre of the liver above the porta hepatis and image analysis was performed as described previously^14^. Scanning sequences included shortened modified Look-Locker inversion recovery (ShMOLLI) T1 and multi-echo spoiled-gradient-echo T2* acquisition. Three 15 mm circular regions of interest were identified as representative parenchymal tissue, and from which the following summary statistics were calculated: proton density fat fraction (PDFF), liver iron concentration and iron-corrected T1 (cT1). PDFF is a reliable measure of liver fat calculated with water-fat separation masks, with PDFF > 5% indicative of fatty liver disease^42^. Liver iron concentration is derived from the T2* signal and is a key biomarker of liver health with high levels linked to fibrosis, cirrhosis, and hepatocellular carcinoma^43^. Finally, cT1 is a novel measure, derived from both T1 and T2* and developed as a composite marker of liver fibrosis and inflammation and linked in recent work to adverse liver-related health events^13,14^. The liver imaging metrics have been previously assessed for reproducibility^44^ and have been used to identify liver differences in healthy and mixed cohorts^45^.

### Brain volumes and white matter microstructure

Multi-modal brain MRI was conducted using a Siemens 3 T scanner^40,46^ using three acquisition types; T1-weighted magnetization-prepared rapid acquisition with gradient echo for overall brain segmentation and volumes, T2-weighted fluid-attenuated inversion recovery for white matter lesion detection, and diffusion-weighted imaging acquisition with 100 diffusion-encoding directions over two shells. A range of standardised brain imaging-derived phenotypes were produced via an extensive data processing and quality control pipeline^47^. Further details of brain MRI acquisition and processing are provided in Supplementary Table 8. In the current study, we included both macroscale and microscale measures of brain structure^48^. At the macroscale, we examined overall brain volume, grey matter volume, and peripheral cortical grey matter volume^49^ which were normalised for head size. Volume of white matter hyperintensities (WMH) was included as a measure of incipient cerebral small vessel disease burden previously linked to neurodegenerative disease^50,51^. At the microscale, we selected intracellular volume fraction (ICVF), and isotropic volume fraction (ISOVF) derived from state-of-the-art diffusion MRI analysis known as neurite orientation dispersion and density imaging (NODDI)^52^. While diffusion analyses have traditionally reported mean diffusivity and fractional anisotropy (FA) as key metrics, NODDI measures were specifically developed to overcome the tendency for the FA signal to be conflated with criss-crossing or complexity in the underlying fibre architecture^52^. Therefore, we chose to follow several recent studies^53,54^ wherein NODDI metrics provided a clearer view of the underlying tissue in terms of intracellular and extracellular water. ICVF refers to the proportion of restricted movement of water diffusion, with higher values of ICVF thought to reflect higher neurite density. ISOVF represents the proportion of water moving in a free-moving or unrestricted motion, with higher values associated with brain oedema, breakdown in myelin and increased permeability of axonal membranes^55^. Diffusion indices across 29 white fibre tracts across the whole brain (excluding the brain stem) were scaled via ordered quantile normalisation^56^ and then averaged to form global measures for ICVF and ISOVF. Details of the fibre tracts included are given in Supplementary Table 9.

### Cardiac measures

Cardiovascular magnetic resonance (CMR) was performed using Siemens 1.5 T scanners, including long-axis cines (horizontal long axis, vertical long axis, left ventricular outflow tract) with a complete short-axis stack and a transverse aortic cine slice at the intersection of the pulmonary trunk and right pulmonary artery. The UK Biobank CMR protocol is available in a separate publication^41^, along with details of image processing and metric derivation^57,58^. We selected CMR measures of cardiovascular structure and function, previously shown to have reliable and intuitively interpretable exposure associations within population cohorts^59^. We selected left ventricular (LV) stroke volume (LVSV) and LV global function index (LV GFI) as measures of LV function. Both have been shown to associate reliably with health and disease indicators; LV GFI, a modified version of LV ejection fraction, which incorporates LV structure, has been shown to have superior prognostic value in population cohorts^12,60^. We included LV mass to LV end-diastolic volume ratio (LVM/LVEDV) as a measure of LV structural remodelling^36^. Higher LVM/LVEDV indicates predominance of a more concentric pattern of LV remodelling, which typically represents an unhealthy structural phenotype. Additionally, we included aortic distensibility, a measure of local aortic bioelastic function, as an indicator of arterial health previously identified as an independent predictor of cardiovascular morbidity and mortality^61,62^.

### Cognitive function

To aid the interpretation of complex brain indices, we calculated their associations with cognitive function. These results are presented alongside the brain analyses as a layer of internal face validity. The battery of cognitive function tests administered by UK Biobank has been previously evaluated for reliability and validity^63,64^. A subset of these were administered at the imaging visit, from which we included fluid intelligence, reaction time (inverted), and prospective memory - based on data availability (>90%) and their broad coverage of cognitive functions (problem-solving, working memory, processing speed). An overall measure of cognitive performance was obtained by calculating the average z-score across the three measures.

### Covariates and confounders

Participants’ sex at birth, date of birth, educational level, and Townsend deprivation score were obtained from the baseline visit. Physical measures (height, weight, waist circumference, and systolic blood pressure) were recorded at the imaging visit. Body surface area and body mass index (BMI) were calculated from height and weight at imaging. Self-reported smoking status, alcohol consumption frequency, and physical activity were recorded at imaging. Physical activity was quantified from responses to the simplified International Physical Activity Questionnaire (IPAQ)^65^ into summed metabolic equivalent (MET)-minutes per week according to UK Biobank guidance^66^. Red blood cell count, total cholesterol and glycated haemoglobin (HbA1c) were taken from blood sampling at baseline. Clinical diagnoses for diabetes, high cholesterol and hypertension were ascertained via a combination of self-report, biochemistry, and linked hospital episode statistics data, with status at the time of imaging. A full listing of UK Biobank fields and codes is given in Supplementary Table 10. In addition, as per published recommendations, we included the following de-confounding variables in brain analyses: head size, imaging site, site by age/sex interactions and de-meaned scanner table coordinates (X, Y, Z and table position)^67^.

### Statistical analysis

Statistical analysis was conducted with R version 4.1.0^68^ and RStudio version 1.4.1717^69^. The main body of the analysis comprised a series of multivariable linear regression analyses to examine pairwise associations between the heart, brain and liver features. All models were adjusted by the following covariates: age, sex, height, diabetes, high cholesterol, hypertension, smoking, physical activity, alcohol consumption, Townsend deprivation index, education, BMI ≥ 30 kg/m^2^, systolic blood pressure, red blood cell count, total cholesterol, and glycated haemoglobin. No missing value imputation was used for the main study variables (i.e., heart, brain and liver metrics). Missing value imputation was used for selected covariates, as detailed in Supplementary Table 11.

Results are presented as standardised beta coefficients with 95% confidence intervals (CIs) and p-values where significance thresholds were adjusted for multiple testing using a false discovery rate of 0.05 across exposure variables across all pairwise models.

Following pairwise analysis, we sought to identify the relationships between heart, brain and liver that persisted when modelled simultaneously, to identify the empirically strongest pathways between organs and potential indirect effects. Features with the greatest number of significant cross-organ links in the pairwise analysis were carried forward into three-organ modelling, including all heart variables, liver fat and liver cT1 and three brain outcomes (total grey matter volume white matter hyperintensities and ISOVF). We modelled simultaneous relationships with path analysis using the *lavaan* package in R^70^. For each brain outcome, liver and heart exposures were modelled together in two forms, (a) Brain ~ heart + liver, Heart ~ liver, and (b) Brain ~ heart + liver, Liver ~ heart, as pictured in Supplementary Fig. 1.

In the first step, all variables were included together in the model, along with all confounders described above as linear (not latent) covariates. The additional imaging confounders were included in paths for the brain outcome. In the second step, non-significant variables were removed one at a time from each formulation, leading to a final model for each outcome in forms (a) and (b). From these, the best fitting model (a or b) was selected based on lowest root mean squared error and highest brain outcome R^2^. Prior to simultaneous modelling, heart and liver variables were orthogonalized with principal components analysis rotation to remove within-organ correlation, while retaining more than 90% of the original signal. Details of each within-organ adjustment are shown in Supplementary Table 12. In supplementary analyses, path analysis was also used to simultaneously relate liver features to heart features.

### Reporting summary

Further information on research design is available in the Nature Portfolio Reporting Summary linked to this article.

## Supplementary information

Supplementary Information

Reporting Summary

## Data Availability

This research was conducted using the UK Biobank resource under access application 59867. UK Biobank will make the data available to all bona fide researchers for all types of health-related research that is in the public interest, without preferential or exclusive access for any persons. All researchers will be subject to the same application process and approval criteria as specified by UK Biobank. For more details on the access procedure, see the UK Biobank website: http://www.ukbiobank.ac.uk/register-apply/. Source data for figures and tables are provided with this paper. Source data are provided with this paper.

## Source data

Source Data

## Code availability

Statistical analysis was conducted with R version 4.1.0^68^ and RStudio version 1.4.1717^69^, making use of the following packages: *tidyverse* (version 1.3.1), *biglm* (version 0.9–2.1), *e1071* (version 1.7–9), *broom* (version 0.8.0), *ggh4x* (version 0.2.1), *mice* (3.14.0), *DiagrammeR* (version 1.0.9) and *lavaan* (version 0.6–11). The R code used for this analysis is publicly available at 10.17605/OSF.IO/ZKMEQ.
