# Supplementary material for: Multi-organ imaging demonstrates the heart-brain-liver axis in UK Biobank participants
Source: Nat Commun. 2022 Dec 21;13:7839. doi: 10.1038/s41467-022-35321-2 (PMC9772225; doi:10.1038/s41467-022-35321-2)
Supplement: Supplementary file 1 — Supplementary Information [file 41467_2022_35321_MOESM1_ESM.pdf]

Supplementary Information

Multi-organ imaging demonstrates the heart-brain-liver axis in UK Biobank participants

Supplementary Table 1: Missingness in study variables 2

Supplementary Table 2: Liver – brain associations 3

Supplementary Table 3: Heart - brain associations 4

Supplementary Table 4: Liver - heart associations.. 5

Supplementary Table 5: Simultaneous liver-heart analysis 6

Supplementary Table 6: Lavaan outputs from three-organ network models.......................... 7

Supplementary Table 7: Technical and clinical validity of liver metrics 8

Supplementary Table 8: Magnetic resonance imaging details 9

Supplementary Table 9: White matter tracts included in average ISOVF and ICVF................ 10

Supplementary Table 10: UK Biobank fields for disease classification 11

Supplementary Table 11: Summary of missing data handling in the main data set 13

Supplementary Figure 1: Alternative forms considered for three-organ path analysis.......... 14

Supplementary Table 12: Component loadings 15

Supplementary References.. 16

Supplementary Table 1: Missingness in study variables

	**Whole set (n= 30,444)**		**Liver subset (n= 15,097)**		**Three-organ set (n= 6,865)**	
**Heart-brain-liver variables**	**Present**	**Missing**	**Present**	**Missing**	**Present**	**Missing**
LV GFI	29,863 (98.1)	581 (1.9)	14,654 (97.1)	443 (2.9)	6,865 (100.0)	-
LVM/LVEDV	29,863 (98.1)	581 (1.9)	14,654 (97.1)	443 (2.9)	6,865 (100.0)	-
LVSVi	29,863 (98.1)	581 (1.9)	14,654 (97.1)	443 (2.9)	6,865 (100.0)	-
AoD	23,079 (75.8)	7,365 (24.2)	11,453 (75.9)	3,644 (24.1)	6,865 (100.0)	-
Liver fat (PDFF)	15,061 (49.5)	15,383 (50.5)	15,061 (99.8)	36 (0.2)	6,865 (100.0)	-
Liver iron	15,077 (49.5)	15,367 (50.5)	15,077 (99.9)	20 (0.1)	6,865 (100.0)	-
Liver cT1 (ms)	11,485 (37.7)	18,959 (62.3)	11,485 (76.1)	3,612 (23.9)	6,865 (100.0)	-
Total brain volume	29,127 (95.7)	1,317 (4.3)	13,780 (91.3)	1,317 (8.7)	6,865 (100.0)	-
Grey matter volume	29,127 (95.7)	1,317 (4.3)	13,780 (91.3)	1,317 (8.7)	6,865 (100.0)	-
White matter hyperintensities	27,954 (91.8)	2,490 (8.2)	13,291 (88.0)	1,806 (12.0)	6,865 (100.0)	-
ICVF	27,148 (89.2)	3,296 (10.8)	12,760 (84.5)	2,337 (15.5)	6,865 (100.0)	-
ISOVF	27,148 (89.2)	3,296 (10.8)	12,760 (84.5)	2,337 (15.5)	6,865 (100.0)	-

Supplementary Table 1: LVSVi = left ventricular stroke volume indexed to body surface area, LV GFI = left ventricular global function index, LVM/LVEDV = left ventricular mass to volume ratio (left ventricular mass / left ventricular end-diastolic volume), AoD = aortic distensibility. PDFF = proton density fat fraction, cT1 = corrected T1 relaxation time, ICVF = intracellular volume fraction, ISOVF = isotropic volume fraction. Source data are provided as a Source Data file.

Supplementary Table 2: Liver – brain associations

		Total brain volume	Grey matter volume	Neurite density (ICVF)	White matter hyperintensities (WMH)	Free-water fraction (ISOVF)
Cognitive performance	Std. beta	0.042*	0.041*	0.052*	-0.047*	-0.025*
N = 25,280 - 27,113	95% CI	[0.032, 0.052]	[0.032, 0.050]	[0.039, 0.064]	[-0.057, -0.036]	[-0.036, -0.013]
	p-value	1.00x10-15	3.97x10-19	4.94x10-16	1.89x10-17	2.18x10-5
	n	27,113	27,113	25,280	25,983	25,280
Liver fat (PDFF, per SD)	Std. beta	-0.019*	-0.025*	0.036*	0.023*	0.019*
N = 12,727 - 13,747	95% CI	[-0.035, -0.003]	[-0.039, -0.011]	[0.016, 0.055]	[0.006, 0.039]	[0.002, 0.037]
	p-value	0.0234	6.32x10-4	3.42x10-4	0.0067	0.0308
	n	13,747	13,747	12,727	13,258	12,727
Fatty liver (PDFF > 5%)	Std. beta	-0.032	-0.040*	0.037	0.033	0.042*
N = 12,727 - 13,747	95% CI	[-0.068, 0.004]	[-0.072, -0.009]	[-0.005, 0.080]	[-0.003, 0.069]	[0.004, 0.080]
	p-value	0.0793	0.0113	0.0876	0.0704	0.032
	n	13,747	13,747	12,727	13,258	12,727
Liver cT1 (per SD increase)	Std. beta	-0.017	-0.029*	-0.029*	0.040*	0.040*
N =9,649 - 10,403	95% CI	[-0.034, 0.000]	[-0.044, -0.013]	[-0.050, -0.008]	[0.022, 0.057]	[0.022, 0.058]
	p-value	0.0565	2.72x10-4	0.0062	8.53x10-6	1.86x10-5
	n	10,403	10,403	9,649	10,138	9,649
cT1 >= 750 ms	Std. beta	-0.031	-0.063*	-0.026	0.073*	0.065*
N =9,649 - 10,403	95% CI	[-0.076, 0.015]	[-0.103, -0.023]	[-0.080, 0.028]	[0.027, 0.119]	[0.017, 0.113]
	p-value	0.1843	0.002	0.3439	0.0017	0.0076
	n	10,403	10,403	9,649	10,138	9,649
Liver iron (per SD)	Std. beta	-0.015	-0.021*	0.038*	0.005	0.012
N = 12,741 - 13,761	95% CI	[-0.029, -0.001]	[-0.033, -0.008]	[0.022, 0.055]	[-0.009, 0.019]	[-0.003, 0.027]
	p-value	0.0402	8.91x10-4	6.53x10-6	0.512	0.11
	n	13,761	13,761	12,741	13,273	12,741

Supplementary Table 2: Entries are standardised beta coefficients, 95% confidence intervals and p-values from linear regression models. Each cell represents one model, with the brain variable placed as the outcome/response and the liver variable placed as the exposure/predictor. Models are adjusted by age, sex, diabetes, hypertension, BMI ≥ 30 kg/m^2, high cholesterol, smoking, physical activity, alcohol consumption, deprivation, educational level, red blood count, total cholesterol, glycosylated haemoglobin, and imaging confounders (including head size, imaging site, scanner coordinates, date of scanning and interactions). P-values are from two-sided T-tests for each coefficient. An asterisk indicates significance after adjustment for multiple testing with a false discovery rate of 5%. PDFF = proton density fat fraction, cT1 = corrected T1 relaxation time, ICVF = intracellular volume fraction, ISOVF = isotropic volume fraction. Source data are provided as a Source Data file.

Supplementary Table 3: Heart - brain associations

		Total brain volume	Grey matter volume	Neurite density (ICVF)	White matter hyperintensities (WMH)	Free-water fraction (ISOVF)
**Cognitive**	Std. beta	0.042*	0.041*	0.052*	-0.047*	-0.025*
**performance**	95% CI	[0.032, 0.052]	[0.032, 0.050]	[0.039, 0.064]	[-0.057, -0.036]	[-0.036, -0.013]
N = 25,280 - 27,113	p-value	1.00x10-15	3.97x10-19	4.94x10-16	1.89x10-17	2.18x10-5
	n	27,113	27,113	25,280	25,983	25,280
**LV Stroke volume**	Std. beta	0.040*	0.031*	0.009	-0.041*	-0.007
**(LVSVi)**	95% CI	[0.030, 0.050]	[0.023, 0.040]	[-0.003, 0.021]	[-0.052, -0.031]	[-0.019, 0.004]
N = 26,653 - 28,561	p-value	7.27x10-16	5.52x10-13	0.1615	4.42x10-15	0.1833
	n	28,561	28,561	26,653	27,438	26,653
**LV Global function**	Std. beta	0.003	0.006	0.038*	-0.050*	-0.033*
**index (LV GFI)**	95% CI	[-0.007, 0.014]	[-0.002, 0.015]	[0.026, 0.051]	[-0.060, -0.039]	[-0.044, -0.021]
N = 26,653 - 28,561	p-value	0.5039	0.1554	1.01x10-9	6.73x10-20	1.68x10-8
	n	28,561	28,561	26,653	27,438	26,653
**LV Mass-to-volume**	Std. beta	-0.003	-0.016*	-0.046*	0.063*	0.045*
**ratio (LVM/LVEDV)**	95% CI	[-0.013, 0.008]	[-0.025, -0.006]	[-0.059, -0.033]	[0.051, 0.074]	[0.033, 0.057]
N = 26,653 - 28,561	p-value	0.6398	0.0011	5.60x10-12	1.92x10-27	3.82x10-13
	n	28,561	28,561	26,653	27,438	26,653
**Aortic distensibility**	Std. beta	0.023*	0.030*	0.060*	-0.098*	-0.067*
**(AoD)**	95% CI	[0.009, 0.037]	[0.018, 0.042]	[0.043, 0.076]	[-0.112, -0.083]	[-0.082, -0.051]
N = 20,610 - 22,080	p-value	0.0011	1.44x10-6	5.02x10-12	8.66x10-40	3.26x10-17
	n	22,080	22,080	20,610	21,166	20,610

Supplementary Table 3: Entries are standardised beta coefficients, 95% confidence intervals and p-values from linear regression models. Each cell represents one model, with the brain variable placed as the outcome/response and the heart variable placed as the exposure/predictor. Models are adjusted by age, sex, diabetes, hypertension, BMI ≥ 30 kg/m^2, high cholesterol, smoking, physical activity, alcohol consumption, deprivation, educational level, red blood count, total cholesterol, glycosylated haemoglobin, and imaging confounders (including head size, imaging site, scanner coordinates, date of scanning and interactions). P-values are from two-sided T-tests for each coefficient. An asterisk indicates significance after adjustment for multiple testing with a false discovery rate of 5%. LVSVi = left ventricular stroke volume indexed to body surface area, LV GFI = left ventricular global function index, LVM/LVEDV = left ventricular mass to volume ratio (left ventricular mass / left ventricular end diastolic volume), AoD = aortic distensibility, ICVF = intracellular volume fraction, ISOVF = isotropic volume fraction. Source data are provided as a Source Data file.

Supplementary Table 4: Liver - heart associations

		LV Stroke volume (LVSVi)	LV Global function index (LV GFI)	LV Mass-to-volume ratio (LVM/LVEDV)	Aortic distensibility (AoD)
**Liver fat**	Std. beta	-0.211*	-0.048*	0.176*	-0.029*
**(PDFF, per SD)**	95% CI	[-0.228, -0.195]	[-0.065, -0.031]	[0.160, 0.192]	[-0.046, -0.012]
N = 11,428 - 14,618	p-value	8.65x10-136	1.76x10-8	1.65x10-107	6.16x10-4
	n	14,618	14,618	14,618	11,428
**Fatty liver**	Std. beta	-0.350*	-0.096*	0.330*	-0.033
**(PDFF > 5%)**	95% CI	[-0.388, -0.313]	[-0.134, -0.059]	[0.295, 0.365]	[-0.071, 0.004]
N = 11,428 - 14,618	p-value	4.43x10-74	4.83x10-7	2.75x10-75	0.0798
	n	14,618	14,618	14,618	11,428
**Liver cT1**	Std. beta	-0.084*	-0.038*	0.097*	0.011
**(per SD increase)**	95% CI	[-0.103, -0.066]	[-0.056, -0.020]	[0.080, 0.113]	[-0.008, 0.031]
N =8,234 - 11,175	p-value	6.21x10-19	3.38x10-5	4.10x10-31	0.2511
	n	11,175	11,175	11,175	8,234
**cT1 >= 750 ms**	Std. beta	-0.174*	-0.070*	0.199*	-0.005
N =8,234 - 11,175	95% CI	[-0.222, -0.125]	[-0.117, -0.022]	[0.157, 0.242]	[-0.055, 0.046]
	p-value	2.96x10-12	0.0038	5.94x10-20	0.852
	n	11,175	11,175	11,175	8,234
**Liver iron**	Std. beta	-0.045*	0.012	0.028*	-0.012
**(per SD)**	95% CI	[-0.060, -0.030]	[-0.002, 0.027]	[0.013, 0.042]	[-0.026, 0.003]
N = 11,438 - 14,635	p-value	4.06x10-9	0.0996	1.27x10-4	0.1086
	n	14,635	14,635	14,635	11,438

Supplementary Table 4: Entries are standardised beta coefficients and p-values from linear regression models. Each beta coefficient represents one model, with the heart variable placed as the outcome/response and the liver variable placed as the exposure/predictor. Models are adjusted by age, sex, diabetes, hypertension, BMI ≥ 30 kg/m^2, high cholesterol, smoking, physical activity, alcohol consumption, deprivation, educational level, red blood count, total cholesterol, glycosylated haemoglobin. P-values are from two-sided T-tests for each coefficient. An asterisk indicates significance after adjustment for multiple testing with a false discovery rate of 5%. PDFF = proton density fat fraction, cT1 = corrected T1 relaxation time, LVSVi = left ventricular stroke volume indexed to body surface area, LV GFI = left ventricular global function index, LVM/LVEDV = left ventricular mass to volume ratio (left ventricular mass / left ventricular end diastolic volume), AoD = aortic distensibility. Source data are provided as a Source Data file.

Supplementary Table 5: Simultaneous liver-heart analysis

Left hand side		Right hand side	Standard. beta	Lower CI	Upper CI	p-value	Fit measures
Reduced LVSV	~	Liver fat (PDFF)	0.227	0.205	0.249	$< 1.00 \times 10^{-250}$	Chi-square =
	~	Liver cT1	0.042	0.022	0.063	5.74×10^{-5}	6.76 (df= 3)
	~	Liver iron	0.051	0.036	0.066	2.91×10^{-11}	RMSEA = 0.021
		(R^2)	0.160				(0.000, 0.025)
Reduced LV GFI	~	Liver fat (PDFF)	0.066	0.044	0.087	2.34×10^{-9}	TLI = 0.994
	~	Liver cT1	0.021	0.001	0.042	0.0415	N = 8,165
		(R^2)	0.190				
LVM/LVEDV	~	Liver fat (PDFF)	0.185	0.166	0.204	$< 1.00 \times 10^{-250}$	
	~	Liver cT1	0.054	0.036	0.072	4.39×10^{-9}	
	~	Liver iron	0.024	0.009	0.039	0.0023	
		(R^2)	0.332				
Aortic stiffening	~	Liver fat (PDFF)	0.030	0.011	0.049	0.0020	
		(R^2)	0.397				

Supplementary Table 5: Entries are standardised beta coefficients and p-values from a single path analysis containing four heart outcomes modelled by all liver exposures entered together. Model was fitted using the *sem* function in the *lavaan* package in R. Initially all liver variables were included for each outcome, then non-significant variables were removed one at a time to arrive at the final model. Liver variables were orthogonalized prior to modelling (see Supplementary Table 12 for details). P-values are from two-sided Z-tests for each coefficient, and p-values were considered significant after adjustment for multiple testing with a 5% false discovery rate. Paths are adjusted by age, sex, diabetes, hypertension, BMI ≥ $30 kg/m^2$, high cholesterol, smoking, physical activity, alcohol consumption, deprivation, educational level, red blood count, total cholesterol, glycosylated haemoglobin. PDFF = proton density fat fraction, cT1 = corrected T1 relaxation time, LVSV = left ventricular stroke volume, LV GFI = left ventricular global function index, LVM/LVEDV = left ventricular mass to volume ratio (left ventricular mass / left ventricular end diastolic volume). Source data are provided as a Source Data file.

Supplementary Table 6: Lavaan outputs from three-organ network models

(showing test paths only – not showing confounder coefficients, N= 6,865)

Model	Equation	Left hand side		Right hand side	Standard. beta	Lower CI	Upper CI	p-value	Fit measures	Reported in Figure 4?
1a	1	Grey matter volume	~	Reduced LVSV	-0.024	-0.043	-0.006	0.0106	Chi-square = 147.70 (df= 9)	Yes
		Grey matter volume	~	Aortic stiffening	-0.038	-0.060	-0.017	4.56x10^{-4}	RMSEA = 0.047 (0.041, 0.054)	
		Grey matter volume	~	Liver ct1	-0.029	-0.046	-0.011	0.0018	TLI = 0.881	
	2	Reduced LVSV	~	Liver fat (PDFF)	0.229	0.206	0.252	< 1.00 x 10^{-250}	R^2 = 0.503	
		Reduced LVSV	~	Liver cT1	0.037	0.015	0.059	9.01x10^{-4}		
1b	1	Grey matter volume	~	Reduced LVSV	-0.025	-0.044	-0.006	0.0112	Chi-square = 529.27 (df= 20)	No
		Grey matter volume	~	Aortic stiffening	-0.038	-0.060	-0.017	4.96x10^{-4}	RMSEA = 0.061 (0.056, 0.065)	
		Grey matter volume	~	Liver cT1	-0.030	-0.048	-0.012	8.86x10^{-4}	TLI = 0.737	
	2	Liver fat (PDFF)	~	LVSVi	0.213	0.189	0.237	< 1.00 x 10^{-250}	R^2 = 0.502	
		Liver fat (PDFF)	~	LV_GFI	-0.038	-0.062	-0.014	0.0021		
		Liver fat (PDFF)	~	LVM/LVEDV	0.152	0.127	0.177	< 1.00 x 10^{-250}		
		Liver fat (PDFF)	~	Aortic stiffening	0.050	0.023	0.076	2.14x10^{-4}		
	3	Liver cT1	~	LVM/LVEDV	0.026	0.001	0.052	0.0457		
2a	1	White matter hyperintensities	~	LV_GFI	0.031	0.009	0.052	0.0046	Chi-square = 480.2 (df= 10)	No
		White matter hyperintensities	~	LVM/LVEDV	0.072	0.050	0.094	1.58x10^{-10}	RMSEA = 0.083 (0.077, 0.089)	
		White matter hyperintensities	~	Aortic stiffening	0.071	0.046	0.095	1.11x10^{-8}	TLI = 0.554	
		White matter hyperintensities	~	Liver cT1	0.032	0.011	0.052	0.0021	R^2 = 0.363	
	2	LVM/LVEDV	~	Liver fat (PDFF)	0.184	0.162	0.207	< 1.00 x 10^{-250}		
		LVM/LVEDV	~	Liver cT1	0.048	0.026	0.069	1.36x10-5		
2b	1	White matter hyperintensities	~	Reduced LV GFI	0.031	0.009	0.052	0.0056	Chi-square = 107.59 (df= 9)	Yes
		White matter hyperintensities	~	LVM/LVEDV	0.072	0.049	0.094	5.91x10^{-10}	RMSEA = 0.040 (0.033, 0.047)	
		White matter hyperintensities	~	Aortic stiffening	0.071	0.046	0.095	1.45x10^{-8}	TLI = 0.855	
		White matter hyperintensities	~	Liver cT1	0.032	0.012	0.052	0.0020	R^2 = 0.363	
	2	Liver ct1	~	LVM/LVEDV	0.026	0.001	0.052	0.0046		
3	1	ISOVF	~	Aortic stiffening	0.068	0.042	0.094	3.41x10^{-7}	Chi-square = 0.00 (df = 0)	Yes
		ISOVF	~	Liver cT1	0.042	0.020	0.064	1.87x10^{-4}	RMSEA = 0.000 , TLI = 1.000	(Both forms reduced
		ISOVF		(R^2)	0.251				R^2 = 0.251	to these paths)

Supplementary Table 6: Final results from multi-organ path analysis for simultaneous liver/heart associations with grey matter volume, white matter hyperintensities and free-water fraction (ISOVF), in the subset with complete cases across three organs (N= 6,865).Path analysis models were fitted using the *sem* function in the *lavaan* package in R. All paths are adjusted by age, sex, height, diabetes, hypertension, high cholesterol, smoking, physical activity, alcohol intake frequency, Townsend deprivation score, education, systolic blood pressure, BMI ≥ 30 kg/m^2, total cholesterol, glycated haemoglobin and red blood cell count. Paths featuring the brain (labelled Equation 1 above) are additionally adjusted by head size, imaging site, scanner coordinates and date of imaging. P-values are from two-sided Z-tests for each coefficient, and p-values were considered significant after adjustment for multiple testing with a 5% false discovery rate. The chi-square in model 3 is zero as this has reduced to a single fitted linear equation. Prior to simultaneous modelling, heart and liver predictors were orthogonalized to remove within-organ correlation (see Supplementary Table 12 for details). Source data are provided as a Source Data file.

Supplementary Table 7: Technical and clinical validity of liver metrics Evidence from mixed/healthy cohorts

A: Measurement and description of liver features in UK Biobank

Study	Sample	Metric(s)	Comment
Wilman et al., (2017)[1]	N= 4,949 UK Biobank	PDFF	Associations with age, BMI, hypertension, T2DM
McKay et al., (2018)[2]	N= 9,108 UK Biobank	PDFF, liver iron	Normal ranges for liver iron
Mojtahed et al.,(2019)[3]	N= 2,816 UK Biobank<br>Subset = 1037 with low risk of NAFLD	PDFF, liver iron, cT1	Normal ranges for cT1

B: Measurement validation of liver features in mixed/healthy cohorts

Study	Sample	Metrics	Comment
Bachtiar et al. (2019)[4]	N= 61, mixed liver conditions= 32, healthy= 29	PDFF, T2*, cT1	All three metrics have good reproducibility across scanner and field strength
Henninger et al. (2021)[5]	N= 369 people with elevated serum ferritin	MRI-derived liver iron	Excellent agreement with biopsy-validated R2*
Hutton et al. (2018)[6]	Phantoms + 4,949 UK Biobank participants	PDFF, T2* (LMS IDEAL)	Excellent agreement of PDFF and T2* across scanners, field strengths and range of values
McDonald et al. (2018)[7]	N= 149 mixed cohort<br>Healthy volunteers = 22	PDFF, T2*, cT1	Good predictive accuracy for all three metrics

C: Epidemiological studies using liver metrics in mixed/healthy cohorts

Study	Sample	Metric(s)	Comment
Harrison et al. (2021)[8]	N= 664, liver steatosis= 244, healthy 420	PDFF, cT1	PDFF and cT1 used to detect NAFLD and fibrosis
Bamberg et al. (2017)[9]	N= 400, healthy = 243, prediabetes = 103, diabetes = 54	PDFF	Prediabetic livers had higher PDFF than normal livers
Rehm et al. (2014)[10]	132 healthy young females	PDFF	Hepatic steatosis detected in 15% of subjects, correlates well with MRS
Qi et al. (2021)[11]	143 liver transplant donors	PDFF	Reliably predicted donor outcomes
Garteiser et al. (2021)[12]	152 obese candidates for bariatric surgery, low alcohol, without serious liver disease (may have NAFLD)	PDFF, confirmed with biopsy	PDFF outperforms transient elastography in diagnosing and grading steatosis
Parisinos et al. (2020)[13]	N= 14,440 UK Biobank participants	cT1	Genetic associations with liver inflammation and scarring
Dennis et al. (2020)[14]	N= 201 COVID positive, healthy controls = 36	PDFF, cT1	Higher liver fat and fibroinflammation in COVID positive patients

Supplementary Table 8: Magnetic resonance imaging details

<table>
<tr><th>Organ</th><th>Sequence</th><th>Acquisition parameters
(as reported by [15])</th><th>Processing, quality control and analysis</th><th>Imaging-derived phenotypes produced</th><th>Key references for full protocol</th></tr>
<tr><td rowspan="3">Liver MRI

Siemens 1.5T MAGNETOM Aera.</td><td>shMOLLI
(shortened modified Look-Locker imaging)</td><td>Resolution: 1.146 x 1.146 x 8,
Matrix: 384 x 288 x 7
TE/TR = 1.93/480.6 ms, α = 35°, R = 2</td><td rowspan="3">Main extraction and analysis via Perspectum's LiverMultiScan™ Discover software [16]

PDFF maps were constructed, using a three-point Dixon technique [17]

Further large-scale phenotyping via a deep learning method [18]</td><td rowspan="3">Proton density fat fraction (PDFF)
Liver iron
Corrected T1 (cT1)</td><td rowspan="3">McKay et al. (2018)[19]
Mojtahed et al. (2019)[20]
Wilman et al. (2017)[21]
Perpectum (2016) [16]</td></tr>
<tr><td>LMS
(LiverMultiScan)</td><td>Resolution: 1.719 x 1.719 x 10
Matrix: 256 x 232 x 6
TE/TR = 1.2 (min) TE/TR = 7.2 (max)/14 ms, α = 5°</td></tr>
<tr><td>ME GRE
(multi-echo spoiled-gradient-echo acquisition)</td><td>Resolution: 2.5 x 2.5 x 6
Matrix: 160 x 160 x 10
TE/TR = 2.38(min)
TE/TR = 23.8(max)/27 ms, α = 20°</td></tr>
<tr><td rowspan="4">Cardiac MRI

Siemens 1.5T MAGNETOM Aera</td><td>LAX
Long axis</td><td>Resolution: 1.9 x 1.9 x 6
Matrix: 210 x 208 x 50
TE/TR = 1.16/32 ms, α = 65°, R = 2</td><td rowspan="4">Imaging-derived markers extracted via a combination of:
• Automatic scanner-provided values
• Expert analysis and segmentation of initial set of 5,000 scans
• Automated image segmentation via deep learning algorithms</td><td rowspan="4">LV stroke volume
LV mass
LV ejection fraction
Aortic distensibility
Left atrial volume
Left atrial ejection fraction
Global longitudinal strain</td><td rowspan="4">Petersen et al. (2013)[22]
Petersen et al. (2016)[23]
Petersen et al. (2017)[22]
Bai et al. (2017)[24]</td></tr>
<tr><td>SAX
Short axis</td><td>Resolution: 1.8 x 1.8 x 8
Matrix: 210 x 208 x 50
TE/TR = 1.1/32 ms, α = 10°, R = 2</td></tr>
<tr><td>shMOLLI</td><td>Resolution: 0.9375 x 0.9375 x 8
Matrix: variable x 384 x 7
TE/TR = 1.073/400 ms, α = 35°, R = 2</td></tr>
<tr><td>Aorta</td><td>Resolution: 1.58 x 1.58 x 6
Matrix: 240 x 196 x 100
TE/TR = 1.17/28 ms, α = 66°, R = 2</td></tr>
<tr><td rowspan="3">Brain MRI

Siemens 3.0T Skyra scanner</td><td>T1 MPRAGE
(magnetization-prepared rapid acquisition with gradient echo)</td><td>Resolution: 1.0 x 1.0 x 1.0
Matrix: 256 x 256 x 208
TI/TR = 880/2000 ms, R = 2</td><td rowspan="3">Imaging-derived markers extracted via the FMRIB imaging processing pipeline [25] including:
• Grey and white tissue volumes estimated using a SIENAX-style analysis [26]
• FSL FAST grey matter segmentation [27]
• White matter hyperintensity volumes with BIANCA [28]
• Probabilistic tractography analysis with BEDPOSTx / PROBTRACKx [29,30]
• Diffusion IDP generation with diffusion tensor fitting (DTI) and NODDI [31]</td><td rowspan="3">Total brain volume
Grey matter volume
White matter volume
Volume of white matter hyperintensities

Fractional anisotropy (FA)
Mean diffusivity (MD)
Intracellular volume fraction (ICVF)
Isometric volume fraction (ISOVF)
Orientation dispersion (OD)</td><td rowspan="3">Smith, Alfaro-Almagro and Miller (2020)[32]
Miller et al. (2016) [33]
Alfaro-Amagro et al. (2018)[25]
Smith et al. (2004)[34]
Zhang et al (2012) [31]</td></tr>
<tr><td>T2 FLAIR
{fluid-attenuated inversion recovery)</td><td>Resolution: 1.0 x 1.0 x 1.05
Matrix: 256 x 256 x 192
TI/TR = 1800/5000 ms, R = 2</td></tr>
<tr><td>Diffusion MRI</td><td>Resolution: 2.0 x 2.0 x 2.0
Matrix: 104 x 104 x 72
TR = 3600 ms, 50 directions/shell,
b = 0, 1000, 2000 s/mm2, α = 51°,
MB = 3</td></tr>
</table>

Supplementary Table 8: Acquisition parameters are reproduced from Littlejohns et al. (2020)[1].T= Tesla, TR= repetition time, TE= echo time, LV = left ventricular, FMRIB = Functional Magnetic Resonance Imaging of the Brain. For additional information on algorithm acronyms please refer to the specified reference.

Supplementary Table 9: White matter tracts included in average ISOVF and ICVF

Group	Cluster	White fibre tract
**Inferior group**	External capsule and uncinate	External capsule
		Uncinate fasciculus
	Inferior pathways	Inferior fronto occipital fasciculus
		Inferior longitudinal fasciculus
		Posterior thalamic radiation
		Sagittal stratum
**Superior group**	Medial / capsules	Acoustic radiation
		Posterior limb of internal capsule
		Retrolenticular part of internal capsule
	Superior pathways	Posterior corona radiata
		Superior corona radiata
		Superior longitudinal fasciculus
		Superior thalamic radiation
**Posterior corpus callosum**		Forceps major
		Splenium of corpus callosum
		Tapetum
**Anterior pathways**		Anterior corona radiata
		Anterior limb of internal capsule
		Anterior thalamic radiation
		Forceps minor
		Superior fronto occipital fasciculus
**Corpus callosum**		Body of corpus callosum
		Genu of corpus callosum
**Cingulum gyrus**		Cingulate gyrus part of cingulum
		Cingulum cingulate gyrus
**Fornix**		Fornix
		Fornix cres/stria terminalis
**Cingulum parahippocampus**		Cingulum hippocampus
		Parahippocampal part of cingulum

Supplementary Table 10: UK Biobank fields for disease classification

Source	Field ID and/or code (if applicable)	Description
**Diabetes**		
Diagnosed by doctor	2443	Diabetes diagnosed by doctor
	2976	Age diabetes diagnosed by doctor
Self-report	20002	diabetes
		type 1 diabetes
		type 2 diabetes
ICD10	E10	Type 1 diabetes mellitus
	E11	Type 2 diabetes mellitus
	E13	Other specified diabetes mellitus
	E14	Unspecified diabetes mellitus
	G590	Diabetic mononeuropathy
	G632	Diabetic polyneuropathy
	H280	Diabetic cataract
	H360	Diabetic retinopathy
	M142	Diabetic arthropathy
	N083	Glomerular disorders in diabetes mellitus
ICD10	O240	Diabetes mellitus in pregnancy: Pre-existing type 1 diabetes mellitus
	O241	Diabetes mellitus in pregnancy: Pre-existing type 2 diabetes mellitus
	O243	Diabetes mellitus in pregnancy: Pre-existing diabetes mellitus, unspecified
	O244	Diabetes mellitus arising in pregnancy
	O249	Diabetes mellitus in pregnancy, unspecified
	Y423	Insulin and oral hypoglycaemic [antidiabetic] drugs
First occurrences	130706	Date E10 first reported (insulin-dependent diabetes mellitus)
	130708	Date E11 first reported (non-insulin-dependent diabetes mellitus)
	130712	Date E13 first reported (other specified diabetes mellitus)
	130714	Date E14 first reported (unspecified diabetes mellitus)
Medications	3	Insulin
Biochemistry	30750	Glycated haemoglobin (HbA1c) >48
ICD9	250	Diabetes mellitus
**High cholesterol**		
Self-report	20002	high cholesterol
ICD10	E780	Pure hypercholesterolaemia
	E782	Mixed hyperlipidaemia
	E783	Hyperchylomicronaemia
	E784	Other hyperlipidaemia
	E785	Hyperlipidaemia, unspecified

First occurrences	130814	Date E78 first reported (disorders of lipoprotein metabolism and other lipidaemias)
Medications	1	Cholesterol lowering medication
Biochemistry	30690	Total cholesterol > 7
**Hypertension**		
Self-report	20002	essential hypertension
	20002	hypertension
ICD10	I10	Essential (primary) hypertension
Medications	2	Blood pressure medication
First occurrences	131286	Date I10 first reported (essential (primary) hypertension)
Diagnosed by doctor	6150: 4	High blood pressure
	2966	Age high blood pressure diagnosed

Supplementary Table 10: Sources for ICD10 are Field IDs 41270, 41280, 41259 and 41234.Sources for ICD9 are Field IDs 41271, 41281, 41259 and 41234.Where a 3-digit ICD code is given, this includes all 4-digit subcodes (for example, E10 includes E100, E101 etc.)

Supplementary Table 11: Summary of missing data handling in the main data set
(N= 30,444)

**Variable**	**Rows**	**%**	**Method**
BMI	804	2.6%	Forward projection from baseline [1]
Height	748	2.5%	Forward projection from baseline [1]
Systolic blood pressure	2,723	8.9%	Forward projection from baseline [1]
Total cholesterol	1,394	4.6%	MICE within biochemistry set [2]
HbA1c	1,575	5.2%	MICE within biochemistry set [2]
Red blood cell count	836	2.7%	MICE within biochemistry set [2]
Alcohol intake frequency	230	0.8%	Forward projection from baseline, and remaining gaps filled with mean [3]
Physical activity	250	0.8%	Forward projection from baseline, and remaining gaps filled with mean [3]
Education	41	0.1%	Replaced with the mean [4]
Cognitive function	629	2.1%	Replaced with the mean [4]

Method details

[1] Target variable was present at the baseline measurement but missing at time of imaging. In these cases, a linear model was constructed based on all data (the full 500,000 UKB set) relating, for example:

Weight_at_imaging ~ Weight at baseline + age + sex + age x sex + days between baseline date and imaging date.

Then the fitted linear estimate was used to impute the value where missing.

[2] MICE (multiple imputation by chained equations) was performed using the *mice* package in R, across the set of well-populated biochemistry fields, with the full set of UKB data, within a set of rows such that all variables were less than 6% missing, with the addition of age, sex, smoking, BMI, alcohol intake and Townsend deprivation score added to the multiple imputation equations. MICE was run with 7 iterations to produce a single data set.

[3] Same process as with method [1] but there were still a small number of residual missing values (missing at both baseline and imaging) which were replaced with the mean.

[4] A very small number of missing values were replaced with the mean.

Supplementary Figure 1: Alternative forms considered for three-organ path analysis

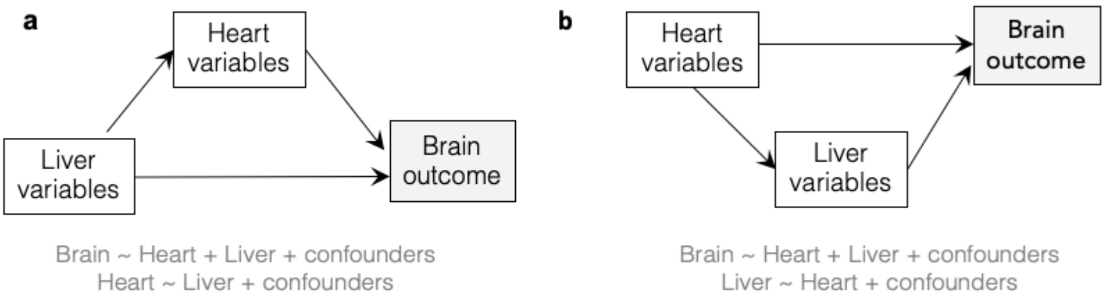

Supplementary Figure 1: Simultaneous heart-brain-liver relationships were investigated using three-organ path analysis in two alternative forms **a** and **b**. In both forms, heart variables are included together (reduced left ventricular stroke volume, reduced left ventricular global function index, increased mass-to-volume ratio and aortic stiffening) and liver variables are included together (liver fat and liver cT1) for a single brain outcome at a time. Brain outcomes studied were grey matter volume, white matter hyperintensities and free-water fraction.

Supplementary Table 12: Component loadings show the correlation between the raw heart/liver features and their principal component counterparts.

	Rotated components				
**Raw liver measures**	**Liver fat**	**Liver iron**	**Liver cT1**		**N**
Liver fat (PDFF)	**0.933**	0.145	0.330		11,452
Liver iron	0.117	**0.991**	-0.069		
Liver cT1	0.320	-0.083	**0.944**		
**Raw heart measures**	**LVM/ LVEDV**	**Aortic stiffening**	**Reduced LVSV**	**Reduced LV GFI**	**N**
LVM/LVEDV	**0.954**	0.101	0.154	0.235	22,798
Aortic stiffening	0.090	**0.993**	0.077	0.022	
Reduced LVSV	0.152	0.085	**0.961**	0.216	
Reduced LV GFI	0.243	0.024	0.227	**0.943**	

Supplementary Table 12: Principal components analysis was performed for each organ, with the number of retained components equal to the number of input variables. Set sizes was determined by complete rows available for each organ. Varimax rotation was applied. For easier interpretability, three original heart variables were reversed so that all heart/liver variables would share the same directionality – these are: aortic stiffening = aortic distensibility * -1, reduced LV stroke volume = LV stroke volume * -1, and reduced LV GFI = LV GFI * -1. LV= left ventricular, SV = stroke volume, GFI = global function index, PDFF= proton density fat fraction, LVM/LVEDV = left ventricular mass to volume ratio. Source data are provided as a Source Data file.

Supplementary References

1. Wilman, H. R. *et al.* Characterisation of liver fat in the UK Biobank cohort. *PLoS ONE* **12**, e0172921 (2017).
2. McKay, A. *et al.* Measurement of liver iron by magnetic resonance imaging in the UK Biobank population. *PLOS ONE* **13**, e0209340 (2018).
3. Mojtahed, A. *et al.* Reference range of liver corrected T1 values in a population at low risk for fatty liver disease—a UK Biobank sub-study, with an appendix of interesting cases. *Abdom. Radiol.* **44**, 72–84 (2019).
4. Bachtiar, V. *et al.* Repeatability and reproducibility of multiparametric magnetic resonance imaging of the liver. *PloS One* **14**, e0214921 (2019).
5. Henninger, B. *et al.* Performance of different Dixon-based methods for MR liver iron assessment in comparison to a biopsy-validated R2* relaxometry method. *Eur. Radiol.* **31**, 2252–2262 (2021).
6. Hutton, C., Gyngell, M. L., Milanesi, M., Bagur, A. & Brady, M. Validation of a standardized MRI method for liver fat and T2* quantification. *PLOS ONE* **13**, e0204175 (2018).
7. McDonald, N. *et al.* Multiparametric magnetic resonance imaging for quantitation of liver disease: a two-centre cross-sectional observational study. *Sci. Rep.* **8**, 9189 (2018).
8. Harrison, S. A. *et al.* Prospective evaluation of the prevalence of non-alcoholic fatty liver disease and steatohepatitis in a large middle-aged US cohort. *J. Hepatol.* **75**, 284–291 (2021).
9. Bamberg, F. *et al.* Subclinical Disease Burden as Assessed by Whole-Body MRI in Subjects With Prediabetes, Subjects With Diabetes, and Normal Control Subjects From the General Population: The KORA-MRI Study. *Diabetes* **66**, 158–169 (2016).

10. Rehm, J. L. *et al.* Proton density fat-fraction is an accurate biomarker of hepatic steatosis in adolescent girls and young women. *Eur. Radiol.* **25**, 2921–2930 (2015).
11. Qi, Q. *et al.* Magnetic resonance imaging-derived proton density fat fraction (MRI-PDFF) is a viable alternative to liver biopsy for steatosis quantification in living liver donor transplantation. *Clin. Transplant.* **35**, e14339 (2021).
12. Garteiser, P. *et al.* Prospective comparison of transient elastography, MRI and serum scores for grading steatosis and detecting non-alcoholic steatohepatitis in bariatric surgery candidates. *JHEP Rep. Innov. Hepatol.* **3**, 100381 (2021).
13. Parisinos, C. A. *et al.* Genome-wide and Mendelian randomisation studies of liver MRI yield insights into the pathogenesis of steatohepatitis. *J. Hepatol.* **73**, 241–251 (2020).
14. Dennis, A. *et al.* Multiorgan impairment in low-risk individuals with post-COVID-19 syndrome: a prospective, community-based study. *BMJ Open* **11**, e048391 (2021).
15. Littlejohns, T. J. *et al.* The UK Biobank imaging enhancement of 100,000 participants: rationale, data collection, management and future directions. *Nature Communications* vol. 11 1–12 Preprint at https://doi.org/10.1038/s41467-020-15948-9 (2020).
16. Perspectum. LiverMultiScan | Perspectum. https://perspectum.com/products/livermultiscan (2016).
17. Glover, G. H. Multipoint dixon technique for water and fat proton and susceptibility imaging. *J. Magn. Reson. Imaging* **1**, 521–530 (1991).
18. Irving, B. *et al.* Deep Quantitative Liver Segmentation and Vessel Exclusion to Assist in Liver Assessment. *Commun. Comput. Inf. Sci.* **723**, 663–673 (2017).
19. McKay, A. *et al.* Measurement of liver iron by magnetic resonance imaging in the UK Biobank population. *PLOS ONE* **13**, e0209340 (2018).

20. Mojtahed, A. *et al.* Reference range of liver corrected T1 values in a population at low risk for fatty liver disease—a UK Biobank sub-study, with an appendix of interesting cases. *Abdom. Radiol.* **44**, 72–84 (2019).
21. Wilman, H. R. *et al.* Characterisation of liver fat in the UK Biobank cohort. *PLoS ONE* **12**, 1–14 (2017).
22. Petersen, S. E. *et al.* Imaging in population science: Cardiovascular magnetic resonance in 100,000 participants of UK Biobank - Rationale, challenges and approaches. *J. Cardiovasc. Magn. Reson.* **15**, (2013).
23. Petersen, S. E. *et al.* UK Biobank's cardiovascular magnetic resonance protocol. *J. Cardiovasc. Magn. Reson.* **18**, (2016).
24. Bai, W. *et al.* Automated cardiovascular magnetic resonance image analysis with fully convolutional networks 08 Information and Computing Sciences 0801 Artificial Intelligence and Image Processing. *J. Cardiovasc. Magn. Reson.* **20**, (2018).
25. Alfaro-Almagro, F. *et al.* Image processing and Quality Control for the first 10,000 brain imaging datasets from UK Biobank. *NeuroImage* **166**, 400–424 (2018).
26. Smith, S. M. Fast robust automated brain extraction. *Hum. Brain Mapp.* **17**, 143–155 (2002).
27. Zhang, Y., Brady, M. & Smith, S. Segmentation of brain MR images through a hidden Markov random field model and the expectation-maximization algorithm. *IEEE Trans. Med. Imaging* **20**, 45–57 (2001).
28. Griffanti, L. *et al.* BIANCA (Brain Intensity AbNormality Classification Algorithm): A new tool for automated segmentation of white matter hyperintensities. *NeuroImage* **141**, 191–205 (2016).

29. Behrens, T. E. J., Berg, H. J., Jbabdi, S., Rushworth, M. F. S. & Woolrich, M. W. Probabilistic diffusion tractography with multiple fibre orientations: What can we gain? *NeuroImage* **34**, 144–155 (2007).
30. De Groot, M. *et al.* Improving alignment in Tract-based spatial statistics: Evaluation and optimization of image registration. *NeuroImage* **76**, 400–411 (2013).
31. Zhang, H., Schneider, T., Wheeler-Kingshott, C. A. & Alexander, D. C. NODDI: Practical in vivo neurite orientation dispersion and density imaging of the human brain. *NeuroImage* **61**, 1000–1016 (2012).
32. Smith, S. M., Alfaro-Almagro, F. & Miller, K. L. UK Biobank - Brain Imaging Documentation. *biobank.ctsu.ox.ac.uk/crystal/docs/brain_mri.pdf*.
33. Miller, K. L. *et al.* Multimodal population brain imaging in the UK Biobank prospective epidemiological study. *Nat. Neurosci.* **19**, 1523–1536 (2016).
34. Smith, S. M. *et al.* Advances in functional and structural MR image analysis and implementation as FSL. *NeuroImage* **23**, S208–S219 (2004).
