# Supplementary material for: Multi-organ imaging demonstrates the heart-brain-liver axis in UK Biobank participants
Source: Nat Commun. 2022 Dec 21;13:7839. doi: 10.1038/s41467-022-35321-2 (PMC9772225; doi:10.1038/s41467-022-35321-2)
Supplement: Supplementary file 2 — Reporting Summary [file 41467_2022_35321_MOESM2_ESM.pdf]

nature portfolio

Corresponding author(s): Zahra Raisi-Estabragh
Last updated by author(s): Nov 2, 2022

Reporting Summary

Nature Portfolio wishes to improve the reproducibility of the work that we publish. This form provides structure for consistency and transparency in reporting. For further information on Nature Portfolio policies, see our Editorial Policies and the Editorial Policy Checklist.

Statistics

For all statistical analyses, confirm that the following items are present in the figure legend, table legend, main text, or Methods section.

n/a	Confirmed	
☐	☒	The exact sample size (*n*) for each experimental group/condition, given as a discrete number and unit of measurement
☐	☒	A statement on whether measurements were taken from distinct samples or whether the same sample was measured repeatedly
☐	☒	The statistical test(s) used AND whether they are one- or two-sided<br>*Only common tests should be described solely by name; describe more complex techniques in the Methods section.*
☐	☒	A description of all covariates tested
☐	☒	A description of any assumptions or corrections, such as tests of normality and adjustment for multiple comparisons
☐	☒	A full description of the statistical parameters including central tendency (e.g. means) or other basic estimates (e.g. regression coefficient) AND variation (e.g. standard deviation) or associated estimates of uncertainty (e.g. confidence intervals)
☐	☒	For null hypothesis testing, the test statistic (e.g. *F*, *t*, *r*) with confidence intervals, effect sizes, degrees of freedom and *P* value noted<br>*Give P values as exact values whenever suitable.*
☒	☐	For Bayesian analysis, information on the choice of priors and Markov chain Monte Carlo settings
☒	☐	For hierarchical and complex designs, identification of the appropriate level for tests and full reporting of outcomes
☒	☐	Estimates of effect sizes (e.g. Cohen's *d*, Pearson's *r*), indicating how they were calculated

Our web collection on statistics for biologists contains articles on many of the points above.

Software and code

Policy information about availability of computer code

Data collection	No data collection software was used other than the general UK Biobank protocol
Data analysis	Analysis was undertaken using with R version 4.1.0 and RStudio version 1.4.1717, making use of the following packages: tidyverse (1.3.1), biglm (0.9-2.1), e1071 (1.7-9), broom (0.8.0), ggh4x (0.2.1), mice (3.14.0), DiagrammeR (1.0.9) and lavaan (0.6-11). Analysis scripts are available at https://www.doi.org/10.17605/OSF.IO/ZKMEQ

For manuscripts utilizing custom algorithms or software that are central to the research but not yet described in published literature, software must be made available to editors and reviewers. We strongly encourage code deposition in a community repository (e.g. GitHub). See the Nature Portfolio guidelines for submitting code & software for further information.

Data

Policy information about availability of data

All manuscripts must include a data availability statement. This statement should provide the following information, where applicable:
- Accession codes, unique identifiers, or web links for publicly available datasets
- A description of any restrictions on data availability
- For clinical datasets or third party data, please ensure that the statement adheres to our policy

This research was conducted using the UK Biobank resource under access application 59867. UK Biobank will make the data available to all bona fide researchers for all types of health-related research that is in the public interest, without preferential or exclusive access for any per- sons. All researchers will be subject to the same application process and approval criteria as specified by UK Biobank. For more details on the access procedure, see the UK Biobank website: http://

www.ukbiobank.ac.uk/register-apply/. Source data for figures and tables are provided with this paper.

Human research participants

Policy information about studies involving human research participants and Sex and Gender in Research.

Reporting on sex and gender	Self-reported sex at birth was used as the measure for sex in the study.<br>We did not include any sex-stratified analyses in this study.
Population characteristics	The overall data set (30,444) is 52% female, average age is 63 years, and is 18% obese. Proportions of participants with diabetes, hypertension and high cholesterol are 5.7%, 32.4% and 34.6% respectively.
Recruitment	UK Biobank used random sampling from United Kingdom GP registration lists to send invitation letters to participants. (See https://www.ukbiobank.ac.uk/media/gnkeyh2q/study-rationale.pdf for full description). Self-selection bias has meant that the UK Biobank cohort is healthier and less ethnically diverse than the UK population as a whole.
Ethics oversight	This study complies with the Declaration of Helsinki; the work was covered by the ethical approval for UK Biobank studies from the National Health Service (NHS) National Research Ethics Service on 17th June 2011 (Ref 11/NW/0382) and extended on 18 June 2021 (Ref 21/NW/0157) with written informed consent obtained from all participants.

Note that full information on the approval of the study protocol must also be provided in the manuscript.

Field-specific reporting

Please select the one below that is the best fit for your research. If you are not sure, read the appropriate sections before making your selection.

☒ Life sciences ☐ Behavioural & social sciences ☐ Ecological, evolutionary & environmental sciences

For a reference copy of the document with all sections, see nature.com/documents/nr-reporting-summary-flat.pdf

Life sciences study design

All studies must disclose on these points even when the disclosure is negative.

Sample size	Within the UKB cohort, heart and brain imaging was available for 31,174 participants. We excluded 730 (2%) participants with missing covariate data, leaving a maximum analysis sample of 30,444 participants. Heart-brain associations were calculated using this set. A subset of 15,097 participants had at least one piece of liver imaging data. Liver-brain and liver-heart associations were calculated on this set. In three-organ path analyses we use only complete rows across all imaging metrics, which includes 6,865 participants.
Data exclusions	730 participants were excluded due to missing height and/or weight measurements. These measurements are critical for indexing cardiac measures and as input to BMI calculations.
Replication	This is an exploratory, observational study. We are simply reporting associations observed in the data set as a whole.
Randomization	There were no experimental groups in our study
Blinding	Blinding was not necessary as there were no experimental groups in our study

Reporting for specific materials, systems and methods

We require information from authors about some types of materials, experimental systems and methods used in many studies. Here, indicate whether each material, system or method listed is relevant to your study. If you are not sure if a list item applies to your research, read the appropriate section before selecting a response.

Materials & experimental systems

n/a	Involved in the study	
☒	☐	Antibodies
☒	☐	Eukaryotic cell lines
☒	☐	Palaeontology and archaeology
☒	☐	Animals and other organisms
☒	☐	Clinical data
☒	☐	Dual use research of concern

Methods

n/a	Involved in the study	
☒	☐	ChIP-seq
☒	☐	Flow cytometry
☒	☐	MRI-based neuroimaging
